# Dose- and application route-dependent effects of betahistine on behavioral recovery and neuroplasticity after acute unilateral labyrinthectomy in rats

**DOI:** 10.3389/fneur.2023.1175481

**Published:** 2023-07-19

**Authors:** Melissa Antons, Magdalena Lindner, Eva Eilles, Lisa Günther, Astrid Delker, Christina Branner, Anja Krämer, Roswitha Beck, Rosel Oos, Max Wuehr, Sibylle Ziegler, Michael Strupp, Andreas Zwergal

**Affiliations:** ^1^German Center for Vertigo and Balance Disorders, DSGZ, LMU University Hospital, LMU Munich, Munich, Germany; ^2^Department of Nuclear Medicine, LMU University Hospital, LMU Munich, Munich, Germany; ^3^Department of Neurology, LMU University Hospital, LMU Munich, Munich, Germany

**Keywords:** vestibular disorders, acute unilateral vestibulopathy, Menière's disease, neuroimaging, animal models, betahistine

## Abstract

**Introduction:**

Betahistine is widely used for the treatment of various vestibular disorders. However, the approved oral administration route and maximum daily dose are evidently not effective in clinical trials, possibly due to a major first-pass metabolism by monoamine oxidases (MAOs). The current study aimed to test different application routes (i.v./s.c./p.o.), doses, and concurrent medication (with the MAO-B inhibitor selegiline) for their effects on behavioral recovery and cerebral target engagement following unilateral labyrinthectomy (UL) in rats.

**Methods:**

Sixty rats were subjected to UL by transtympanic injection of bupivacaine/arsanilic acid and assigned to five treatment groups: i.v. low-dose betahistine (1 mg/kg bid), i.v. high-dose betahistine (10 mg/kg bid), p.o. betahistine (1 mg/kg bid)/selegiline (1 mg/kg once daily), s.c. betahistine (continuous release of 4.8 mg/day), and i.v. normal saline bid (sham treatment; days 1–3 post-UL), respectively. Behavioral testing of postural asymmetry, nystagmus, and mobility in an open field was performed seven times until day 30 post-UL and paralleled by sequential cerebral [^18^F]-FDG-μPET measurements.

**Results:**

The therapeutic effects of betahistine after UL differed in extent and time course and were dependent on the dose, application route, and selegiline co-medication: Postural asymmetry was significantly reduced on 2–3 days post-UL by i.v. high-dose and s.c. betahistine only. No changes were observed in the intensity of nystagmus across groups. When compared to sham treatment, movement distance in the open field increased up to 5-fold from 2 to 30 days post-UL in the s.c., i.v. high-dose, and p.o. betahistine/selegiline groups. [^18^F]-FDG-μPET showed a dose-dependent rCGM increase in the ipsilesional vestibular nucleus until day 3 post-UL for i.v. high- vs. low-dose betahistine and sham treatment, as well as for p.o. betahistine/selegiline and s.c. betahistine vs. sham treatment. From 1 to 30 days post-UL, rCGM increased in the thalamus bilaterally for i.v. high-dose betahistine, s.c. betahistine, and p.o. betahistine/selegiline vs. saline treatment.

**Discussion:**

Betahistine has the potential to augment the recovery of dynamic deficits after UL if the administration protocol is optimized toward higher effective plasma levels. This may be achieved by higher doses, inhibition of MAO-based metabolism, or a parenteral route. *In vivo* imaging suggests a drug-target engagement in central vestibular networks.

## Introduction

Central vestibular compensation is a process of lesion-induced adaptive brain plasticity that plays a key role in the recovery of symptoms (such as postural and gait imbalance) following acute unilateral peripheral vestibular lesions (1–8). An improved understanding of the underlying mechanisms is needed to define molecular targets for drugs that can augment vestibular compensation and thereby facilitate functional recovery from acute vestibular syndrome (9–11). Previous studies in vestibular animal models suggested betahistine as a promising drug candidate (12–15). Betahistine acts as a weak agonist to H1 receptors and a strong antagonist to H3 receptors in the central nervous system and peripheral vestibular system (16, 17); action on H3 autoreceptors and heteroreceptors leads to an increase of the release of histamine and other transmitters. Important target sites for drug engagement in the brain seem to be the tuberomammillary nucleus in the posterior hypothalamus, the vestibular nuclei, and the inferior olive (18). H1 receptor expression is selectively increased in commissural GABAergic neurons located in the ipsilesional medial vestibular nucleus after unilateral labyrinthectomy in rats, a mechanism that may further promote and facilitate the effect of betahistine on vestibular compensation at the level of the vestibular nuclei (14).

While preclinical models consistently indicate the beneficial effects of betahistine on vestibular compensation, its therapeutic efficacy in patients with acute unilateral vestibulopathy is still a matter of debate. The reasons for this discrepancy are as follows: (1) Different routes of drug application were tested in animal models (i.v. and i.p.) compared to humans (p.o.). It is important to note that 99% of orally ingested betahistine is metabolized in the gastrointestinal tract and liver by MAO-B and MAO-A (19). (2) The dose-dependency of the therapeutic effect was neglected (20). (3) The effect of dose and application route for drug-target engagement in the central vestibular networks was not tested yet. Taken together, further investigations are needed to determine the optimal application route and drug dose for betahistine to augment vestibular compensation and specifically find settings, which will allow an easy translation to patients. In this respect, promising strategies are to increase the dose of betahistine (21), to block its metabolism using MAO-B inhibitors (13, 22), or to apply the drug parenterally to circumvent the first-pass metabolism (15, 23).

The current study was designed along these lines to systematically investigate the effect of betahistine dosage and application route (i.v., s.c., and p.o.) on functional recovery after an acute unilateral vestibular lesion in the rat model and visualize the respective drug-target engagement in central nervous networks *in vivo* by serial [^18^F]-FDG-μPET.

## Materials and methods

### Animals and housing

All animal experiments were approved by the Government of Upper Bavaria and performed in accordance with the guidelines for the use of living animals in scientific studies and the German Law for the Protection of Animals (ROB-55.2-2532.Vet_02-93-16).

Ten–week-old male Sprague-Dawley rats (Charles River, Sulzfeld) with a mean weight of 400 g at the time of surgery were housed two animals per cage in a temperature- and humidity-controlled room with a 12-h light/dark cycle and free access to food and water. All rats were placed in double-decker cages (GR1800, Tecniplast, Germany).

### Experimental procedure

In total, 60 rats were subjected to a chemical unilateral labyrinthectomy (UL) using a transtympanic injection of bupivacaine and arsanilic acid. Seven rats had to be excluded based on predefined criteria (see below), and the remaining 53 rats were distributed to five treatment groups as follows:

- i.v. low-dose betahistine group (*n* = 11): 1 mg/kg body weight of betahistine i.v. bid.
- i.v. high-dose betahistine group (*n* = 9): 10 mg/kg body weight of betahistine i.v. bid.
- p.o. betahistine/selegiline group (*n* = 12): 1 mg/kg body weight of betahistine p.o. bid combined with 1 mg/kg body weight of MAO-B inhibitor selegiline p.o. once daily.
- s.c. betahistine group (*n* = 11): continuous release of 4.8 mg betahistine per day through an osmotic pump implanted subcutaneously.
- sham treatment group (*n* = 10): treatment with normal saline i.v. bid.

Each group received treatment on days 1–3 post-UL. The regional cerebral glucose metabolism was measured by [^18^F]-FDG-μPET on days 1, 3, 7, 15, 21, and 30 post-UL and compared to a baseline scan. In addition, behavioral testing in the open field and clinical scoring (for nystagmus, postural asymmetry, and head tilt) were carried out on days 1, 2, 3, 7, 15, 21, and 30 post-UL following the experimental protocol and timeline of previous studies (24, 25).

### Chemical unilateral labyrinthectomy

A chemical unilateral labyrinthectomy was performed as described earlier (24–27): Perioperative analgesia was ensured by pre-emptive subcutaneous administration of meloxicam (1 mg/kg) s.c. 30 min before the procedure. After the initiation of the anesthesia with 2% isoflurane in O_2_ (1–2 l/min) via a mask, local anesthesia with 0.5% bupivacaine solution (500 μl) was applied s.c. ~1 cm dorsomedially of the ear. A double-sided injection of 2.5 ml of saline solution into the knee fold was applied to stabilize circulation during surgery. For infection prophylaxis, marbofloxacin was administered s.c. at a dosage of 2 mg/kg. The surgical field was opened with a paramedian incision, exposing the lambdoid ridge and the external auditory canal. After opening the external auditory canal anterior to the exit point of the facial nerve, the tympanic membrane was perforated caudally to the hammer shaft with a 26-gauge needle. Afterward, 20% bupivacaine solution (150 μl) was injected into the tympanic cavity. The substance was then repeatedly applied and aspirated to avoid bagging into the Eustachian tube. The same procedure was repeated with 10% arsanilic acid (150 μl), which was previously shown to induce irreversible toxic damage to the primary sensory cells of the inner ear (28). The wound closure was done by skin suture. The analgesic and antibiotic supply was continued postoperatively for a further 3 days by the administration of meloxicam (2 mg/kg) s.c. bid and administration of marbofloxacin (2 mg/kg) s.c. once daily.

### Criteria for exclusion

Animals were excluded from the study if the following symptoms were observed:

- Loss of body weight equal to or more than 20% of the value before UL.
- Ulcer of the cornea, which could occur due to an inadvertent lesion of the facial nerve during UL.
- Bleeding from the tympanic cavity, which could prevent the diffusion of bupivacaine or arsanilic acid into the inner ear.
- Circulatory failure or peracute apnea with lethal consequences.

Based on these criteria, seven animals had to be excluded from the experiment in the following groups: i.v. low-dose betahistine group (*n* = 1), i.v. high-dose betahistine group (*n* = 3), s.c. betahistine group (*n* = 1), and sham treatment group (*n* = 2).

### Instrumental analysis of locomotion and exploration behavior

In all rats, locomotion and spatial exploration behavior were recorded sequentially before UL (baseline) and on days 1, 2, 3, 7, 15, 21, and 30 post-UL in an open field (70 × 70 × 36 cm) using an automated video tracking system mounted above the open field that detects the nose point, center of the body, and tail point (EthoVision^®^ XT 16, Noldus^®^, Netherlands). For each trial, the animals were put into the open field individually and always from the same side and were then allowed to move freely within the arena for 10 min. A black curtain around the open field setup and a defined light source with 45–50 lux ensured optimal control of visual conditions and potential confounding factors. During the experiment, absolute silence was implemented in the room. Only one person was present, who started the trial run on the computer connected to the camera system. The following body points were used for further analysis: tail base, body axis center (center point), and nose tip (nose point).

### Clinical scoring

Signs of vestibular imbalance, namely the postural asymmetry and the nystagmus, were scored as reported previously (27, 29):

- Postural deficits: spontaneous barrel rolling−10 points; barrel rolling evoked by light touch or air-puff—nine points; recumbent position on lesion side without leg support—eight points; some ipsilesional leg support—seven points; moving around on one side or using ipsilesional legs for recumbent support—six points; moving around with bilateral leg support—five points; moving around with occasional falls to the ipsilesional side—four points; moving around leaning toward the ipsilesional side—three points; hardly noticeable asymmetry—two points; postural asymmetry only noticeable when picked up—one point.
- Nystagmus was visually observed with the animal recumbent. In the presence of spontaneous nystagmus, the intensity was scored with either six points (60 beats per min—bpm), seven points (120 bmp), eight points (180 bmp), nine points (240 bmp), or 10 points (300 bmp). In the absence of spontaneous nystagmus at rest, the animal was touched slightly. If a provocation nystagmus was evoked, it was scored one point (60 bmp) to five (300 bmp) points (one point for every 60 bpm).

The clinical scoring was carried out in a single-blinded manner with the experimenter conducting the scoring always blinded for the treatment condition. Clinical scores were documented on a fact sheet with the animal ID, stored until the end of the experiment, and analyzed thereafter on a group level. This procedure was pursued to exclude a potential bias in the clinical rating.

### PET imaging

The animals were kept in a fasting state for 6 h and then anesthetized with 2% isoflurane in O_2_ (1–2 l/min) via a mask. For the application of the tracers, the lateral tail vein was catheterized (24-gauge), and a bolus with 40 MBq of the tracer was injected in 0.5 ml of saline. Thereafter, rats were wakened and allowed to move freely for 25 min to enable an optimized [^18^F]-FDG uptake on naturalistic conditions. Subsequently, anesthesia with 2% isoflurane was induced again, and the animals were positioned on a heating pad in the μPET-CT scanner (Inveon, Siemens, Germany). To avoid any passive movement of the head, its position was fixed using a custom-made head-holder. The [^18^F]-FDG measurements were carried out as static scans with an acquisition from 30 to 60 min p.i. For individual attenuation correction, a transmission (7 min in duration) was performed for each measurement.

### Image processing and statistical analysis

The PET reconstruction procedure was an Ordered Subsets Expectation Maximization (OSEM-3D) algorithm with decay correction, scatter correction, attenuation correction, dead time correction, and sensitivity normalization (Siemens, Germany). The resulting images had 212 × 212 × 235 voxels of 0.4 × 0.4 × 0.4 mm3. A whole-brain normalization was applied in order to achieve comparability between all images. Normalized activity distributions for the [^18^F]-FDG scans were used as a surrogate for the regional cerebral glucose metabolism (rCGM). To delineate regions with significant changes of rGCM following UL, a voxel-wise analysis based on *t*-tests with Bonferroni correction for multiple testing was performed in SPM 8 software (Wellcome Department of Cognitive Neurology, Great Britain) between the i.v. high-dose betahistine group, i.v. low-dose betahistine group, p.o. betahistine/selegiline group, s.c. betahistine osmotic pump group, and sham treatment group, respectively, as well as the i.v. high-dose and i.v. low-dose betahistine group for all imaging time points. A *p*-value of < 0.001 was considered significant as suggested by previous studies (24, 26, 30).

### Statistics

Statistical analysis was performed with IBM SPSS 25 software and Microsoft Excel. Descriptive statistics are reported as mean ± SD. The differential effects of betahistine dosage and application route on functional recovery (open field parameters and clinical scoring of nystagmus and postural asymmetry) after an acute UL were analyzed by mixed linear models with the fixed factors “treatment group” (sham treatment, i.v. low-dose betahistine, i.v. high-dose betahistine, p.o. betahistine/selegiline, and s.c. betahistine osmotic pump) and “time point” post-UL (days 1, 2, 3, 7, 15, 21, and 30). *Post-hoc* adjustments were used to control for multiple comparisons within each model. The results were considered significant if the *p*-value is < 0.05.

## Results

### Effect of treatment regimen on functional recovery from UL in behavioral testing

**Figure 1:**
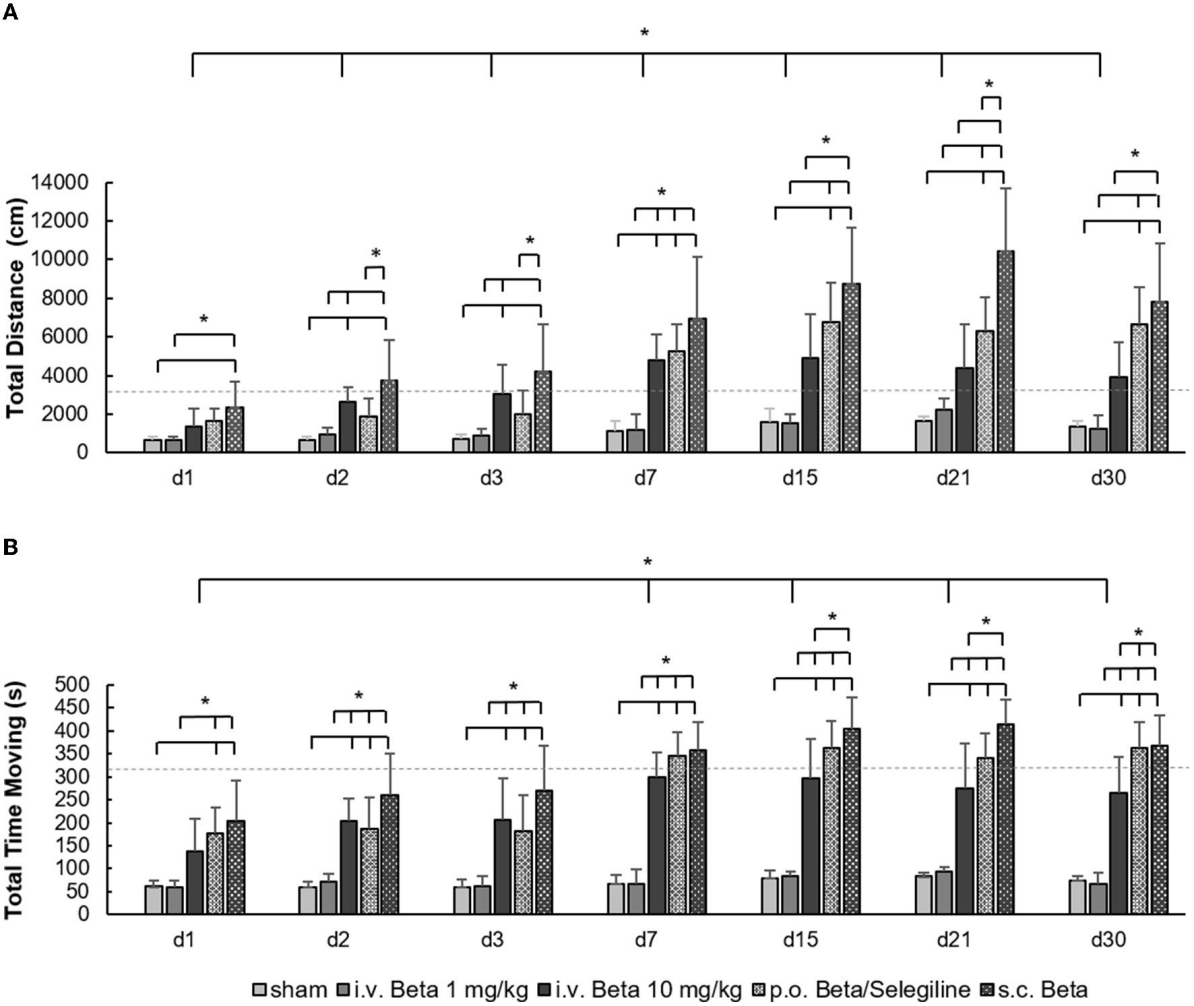
Locomotor parameters in the open field. **(A)** The total amount of the distance moved (in cm) in the open field showed a significant increase in the i.v. high-dose betahistine group, the p.o. betahistine/selegiline group, and the s.c. betahistine osmotic pump group on most examination days post-UL compared to the sham treatment group and i.v. low-dose betahistine group. **(B)** The same pattern was found for the cumulative duration of movement (in s). The baseline data for the respective parameters before UL are depicted as dashed lines as a reference. Beta, betahistine; d, day; cm, centimeter; s, seconds. *p < 0.05, error bars represent +/– SD.

The time point of testing had a significant effect on the total distance moved (*F* = 36.9, *p* < 0.001) and the cumulative time spent moving in the open field (*F* = 26.7, *p* < 0.001), with a general trend of increased mobility above baseline level in the course after UL. Compared to the baseline value before UL, the total distance moved was higher in the s.c. betahistine osmotic pump group, the i.v. high-dose betahistine group, and the p.o. betahistine/selegiline group from days 7 to 30 post-UL. Furthermore, there was a significant effect of the treatment group for the total distance moved (*F* = 78.1, *p* < 0.001) and the cumulative time spent moving (*F* = 207.3, *p* < 0.001). Compared to the sham treatment group, the total distance moved was significantly higher in the s.c. betahistine osmotic pump group for all time points post-UL, in the p.o. betahistine/selegiline group on days 7 to 30 post-UL, and in the i.v. high-dose betahistine group on days 2, 3, and 7 post-UL, while the values of the i.v. low-dose betahistine group did not differ at any time point post-UL (Figure 1A). Rats in the s.c. betahistine, p.o. betahistine/selegiline, and i.v. high-dose betahistine groups spent more time moving than the sham treatment group from day 2 to 30 post-UL (*p* < 0.05), while there was no difference between the i.v. low-dose betahistine group and the sham treatment group. From day 7, the total time moving of the animals in the i.v. high-dose betahistine group equals the baseline level before UL, while the s.c. betahistine osmotic pump group and the p.o. betahistine/selegiline group ranked above baseline (Figure 1B). Individual values per animal, day, and treatment condition are presented as a data supplement (Supplementary Figure 1).

**Figure 2:**
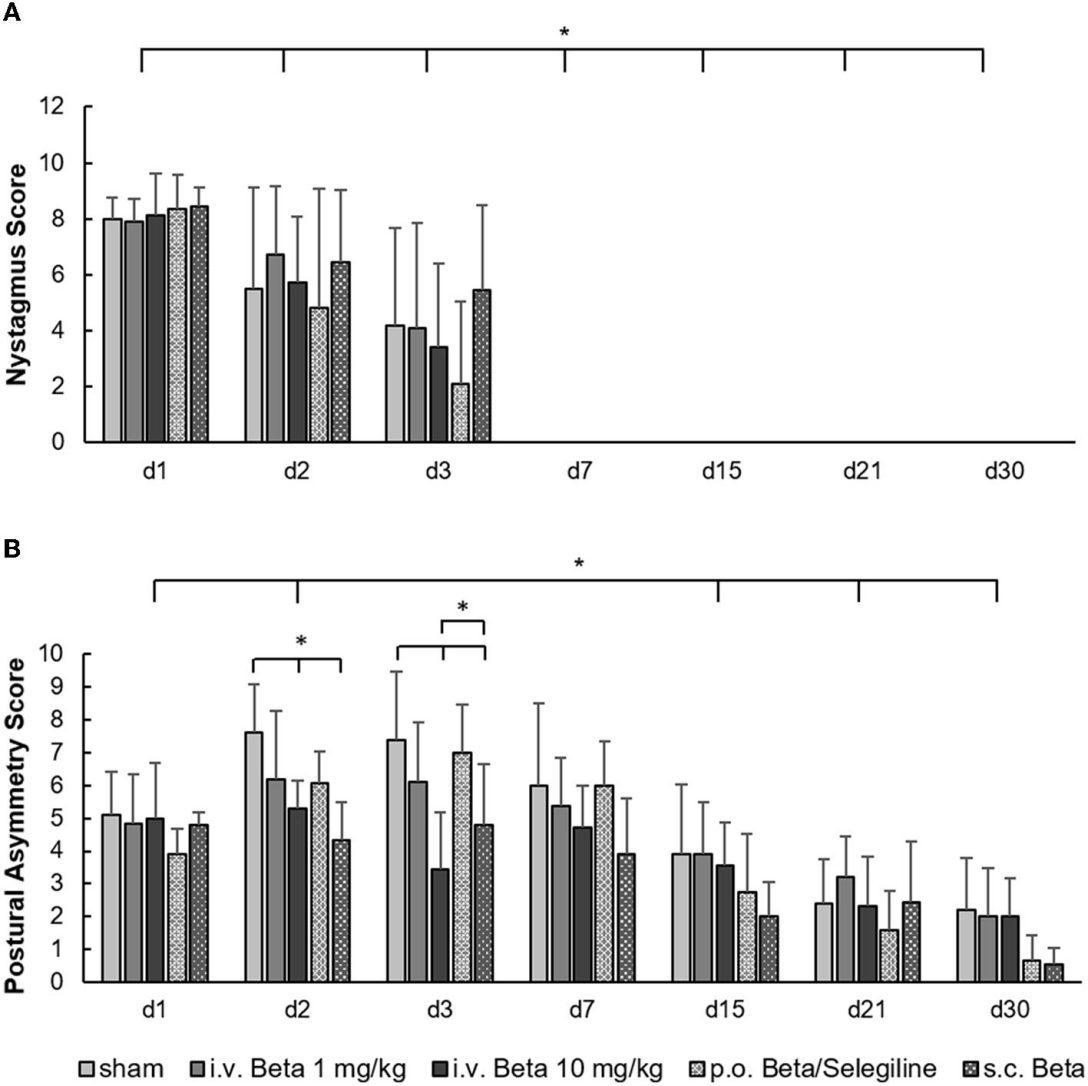
Clinical scoring of nystagmus and postural asymmetry post-UL. **(A)** The nystagmus decreased steadily and disappeared by day 7 post-UL in all groups without a significant effect of either dose or route of betahistine treatment. **(B)** The postural asymmetry scores were significantly improved in the i.v. high-dose betahistine group and the s.c. betahistine osmotic pump group on days 2 and 3 post-UL compared to the sham treatment group. The p.o. betahistine/selegiline group and s.c. betahistine group showed a tendency to lower postural asymmetry on day 30 post-UL (*p* = 0.06). The i.v. low-dose betahistine treatment had no effect on postural scores at any time point. Beta, betahistine; d, day. *p < 0.05, error bars represent +/– SD.

In clinical scoring, a strong nystagmus was present in all groups on day 1 post-UL and decreased steadily, until it disappeared by day 7. Neither treatment had a significant effect on nystagmus intensity (Figure 2A). For postural asymmetry scores, there was a significant effect of the treatment group (*F* = 11.3, *p* < 0.001). Postural asymmetry significantly improved in the i.v. high-dose betahistine group and the s.c. betahistine osmotic pump group relative to the sham treatment group on days 2 and 3 post-UL, and in the p.o. betahistine/selegiline and s.c. betahistine osmotic pump group by tendency vs. sham treatment group (*p* = 0.06, respectively) on day 30 post-UL (Figure 2B). Detailed data per animal, day, and treatment condition are presented as a data supplement (Supplementary Figure 2).

### Brain drug-target engagement depicted by [^18^F]-FDG-μPET

**Figure 3:**
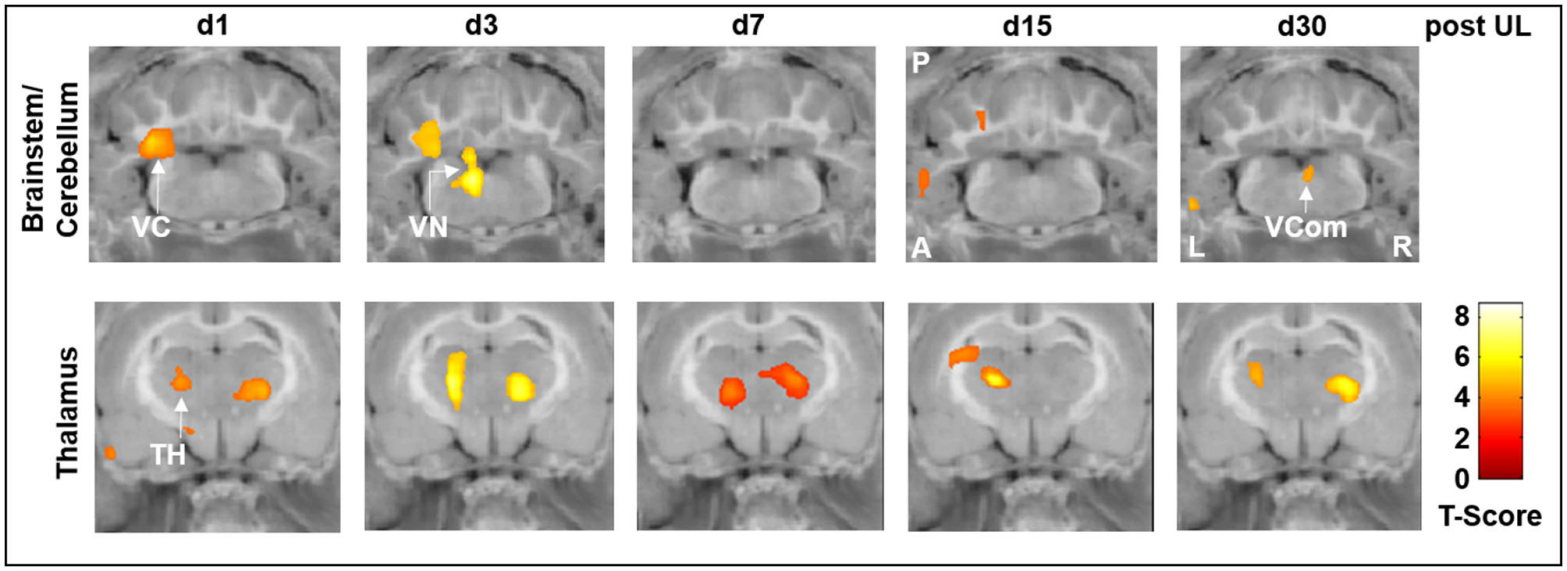
[^18^F]-FDG uptake of the i.v. high-dose betahistine group compared to the sham treatment group. In brainstem–cerebellar networks, the i.v. high-dose betahistine group showed a significant increase in [^18^F]-FDG uptake in the ipsilesional vestibular nucleus (max. day 3 post-UL) and vestibulocerebellum compared to the sham treatment group. In the thalamus, the i.v. high-dose betahistine group had a bilaterally increased [^18^F]-FDG uptake compared to the sham treatment group from day 1 to 30 post-UL. TH, thalamus; UL, unilateral labyrinthectomy; VC, vestibulocerebellum; VCom, vestibular commissure; VN, vestibular nucleus; A, anterior; P, posterior; L, left; R, right; [^18^F]-FDG, [^18^F]-fluorodeoxyglucose.

The serial [^18^F]-FDG-μPET showed a specific target engagement in cerebral vestibular networks across all betahistine treatment groups, which varied in extent as a function of betahistine dose and application route:

Specifically, rCGM in the i.v. high-dose betahistine group was significantly increased in the ipsilesional vestibular nucleus on day 3 and the ipsilesional vestibulocerebellum on days 1 and 3 post-UL, compared to the sham treatment group (Figure 3, top row). Increased activation in the ipsilesional vestibulocerebellum (flocculus) on days 15 and 30 post-UL, as well as in the vestibular commissure on day 30 post-UL, was shown in the i.v. high-dose betahistine group. In the thalamus, rCGM increased on days 1, 3, 7, 15, and 30 post-UL following i.v. high-dose betahistine treatment (Figure 3, bottom row).

**Figure 4:**
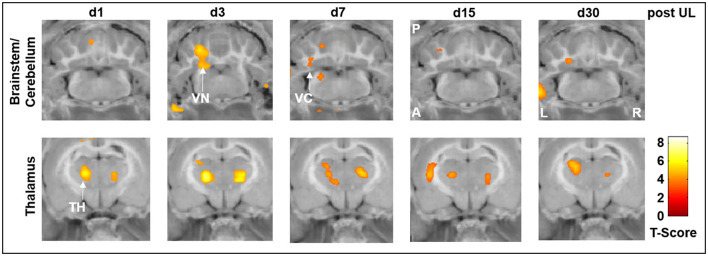
[^18^F]-FDG uptake of the i.v. high-dose betahistine group compared to the i.v. low-dose betahistine group. The i.v. high-dose betahistine group showed an increased effect on [^18^F]-FDG uptake in the ipsilesional vestibular nucleus and vestibulocerebellum (max. days 3–7 post-UL) compared to the i.v. low-dose betahistine group. In the thalamus, the [^18^F]-FDG uptake increased bilaterally from day 1 to 30 post-UL in the higher betahistine dose. TH, thalamus; UL, unilateral labyrinthectomy; VC, vestibulocerebellum; VN, vestibular nucleus; A, anterior; P, posterior; L, left; R, right; [^18^F]-FDG, [^18^F]-fluorodeoxyglucose.

While there were no significant differences in the rCGM between the i.v. low-dose betahistine group and the sham treatment group, comparison of the i.v. high-dose betahistine group vs. the i.v. low-dose betahistine group indicated a dose-dependency of the treatment effect on [^18^F]-FDG uptake in the above-named vestibular hubs. rCGM was higher in the ipsilesional vestibular nucleus on days 3 and 7, and in the ipsilesional vestibulocerebellum on days 3, 7, and 15 post-UL in the i.v. high- vs. low-dose betahistine group (Figure 4, top row). Similarly, bithalamic [^18^F]-FDG uptake was higher from days 1 to 30 post-UL as an effect of the betahistine dose (Figure 4, bottom row).

**Figure 5:**
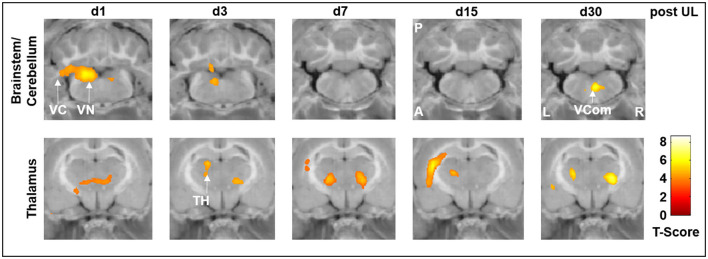
[^18^F]-FDG uptake of the p.o. betahistine/selegiline group compared to the sham treatment group. In the p.o. betahistine/selegiline group, [^18^F]-FDG uptake significantly increased in the ipsilesional vestibular nucleus and vestibulocerebellum on days 1 and 3 post-UL. In the thalamus, the rCGM was higher bilaterally on all days. TH, thalamus; UL, unilateral labyrinthectomy; VC, vestibulocerebellum; VCom, vestibular commissure; VN, vestibular nucleus; A, anterior; P, posterior; L, left; R, right; [^18^F]-FDG, [^18^F]-fluorodeoxyglucose.

In the p.o. betahistine/selegiline group, rCGM was significantly increased compared to sham treatment in the ipsilesional vestibular nucleus and vestibulocerebellum on days 1 and 3 post-UL and in the vestibular commissure on day 30 post-UL (Figure 5, top row). In the thalamus, the rCGM increased bilaterally on days 1, 3, 7, and 30 and ipsilaterally on day 15 p.o. on this treatment regimen (Figure 5, bottom row).

Accordingly, in the s.c. betahistine osmotic pump group, [^18^F]-FDG uptake was increased on days 1 and 3 post-UL in the ipsilesional vestibular nucleus and vestibulocerebellum compared to the sham treatment group (data not shown).

## Discussion

Betahistine has been used since its approval more than 50 years ago for the treatment of different vestibular disorders, ranging from acute unilateral vestibulopathy to Menière's disease. However, clinical evidence for its efficacy is very limited (31, 32). Therefore, there is a high need to reevaluate the basic pharmacokinetic and -dynamic principles underlying the drug action of betahistine in vestibular disorders. The current study in a preclinical rat model of acute unilateral vestibulopathy aimed to test the route- and dose-dependency of betahistine effects and its target engagement in central nervous system networks.

The major findings were the following: (1) Betahistine had beneficial effects mostly on the recovery of dynamic markers of vestibular compensation (i.e., locomotor parameters) and to only a minor extent also of static signs (i.e., postural asymmetry) following acute unilateral vestibular damage. There was no significant effect on the compensation of nystagmus. (2) The treatment effect of betahistine on locomotor parameters critically depended on the route and dose of application. The best effects on locomotor activity were seen with parenteral administration routes or inhibition of the first-pass metabolism by adding the MAO-B inhibitor selegiline. (3) Sequential whole-brain [^18^F]-FDG-μPET imaging showed a dose-dependent engagement of betahistine in plasticity mechanisms occurring at the level of the ipsilesional vestibular nucleus and vestibulocerebellum in the early stage of vestibular compensation. Additional effects were found at the thalamic level, which may reflect secondary mechanisms of multisensory substitution and integration.

### From pharmacokinetics to optimized treatment effects

While a general therapeutic effect of betahistine in acute peripheral vestibular syndrome was indicated by studies in various animal models (33–35), the verdict on the strength of this effect seems to vary (36). The key to understanding the potentially ambiguous effects of betahistine seems to be its specific metabolism and pharmacokinetic properties. Betahistine is a substrate for MAO enzyme types A and B in both the intestine and the liver (19). Consequently, oral administration of betahistine results in a subtotal clearance to its major metabolite 2-pyridyl acetic acid (2-PAA, which is pharmacologically not active in humans) by a first-pass mechanism. Betahistine and 2-PAA reach a plasma peak (t_max_) after oral administration within 1 h at the latest, which indicates rapid absorption from the upper intestinal tract (13). Mean maximal plasma concentrations (C_max_) of betahistine critically depend on the applied dose and the duration of its administration. In cats, a 5-fold increase in maximum plasma levels was found with a 10-times higher oral dose of betahistine on day 1 of administration, which further amplified to a 65-fold increase after 21 days of treatment (13). Accordingly, betahistine plasma levels showed a dose proportionality in healthy controls over a range of 8–24 mg p.o. (20). Blockage of the first-pass metabolism by the MAO-B inhibitor selegiline in the UL cat model resulted in an up to a 20-fold increase in betahistine peak plasma concentration (13). In humans, a phase 1 trial showed an increase in the bioavailability of betahistine by a factor of 100 when combined with selegiline [5 mg/day; (37)]. Based on these assumptions, we would expect an at least 10-fold higher betahistine plasma level for the i.v. high-dose vs. i.v. low-dose group and similarly for the p.o. betahistine/selegiline vs. sham treatment group. However, this has not been proven by explicit plasma analysis in the current study. Parenteral routes of application, such as intranasal, transdermal, or subcutaneous application, have been propagated as an alternative strategy to overcome the first-pass effect (15, 38). However, such drug formulations are not yet available in clinical practice. To summarize current knowledge across species, the biological effects of betahistine on vestibular compensation may depend on the route of application, the therapeutic dose, the duration of treatment, and the modulation of its degradation by MAO enzymes, i.e., finally on its plasma concentration.

In the current study, we tested different application protocols for betahistine against each other to directly compare their therapeutic effect on static vestibular parameters (i.e., nystagmus, postural asymmetry) and dynamic behavior (i.e., mobility in the open field) in the course of vestibular compensation. For postural asymmetry, we found a positive treatment effect of i.v. high-dose betahistine and s.c. betahistine via an osmotic pump against sham treatment on days 2 and 3 post-UL only (Figure 3). This finding is in accordance with previous data from a UL cat model, where betahistine dose-dependently also had an acute effect on postural asymmetry (13). We did not see any change in the intensity of nystagmus across treatment groups, similar to another recent rat study (15). A possible explanation could be the fast and complete compensation of nystagmus by day 7 post-UL, and the relatively rough clinical graduation of nystagmus, which may not be suited to detect subtle differences across groups. Mobility parameters (e.g., distance moved) in the acute stage of the vestibular syndrome (i.e., within the first 3 days post-UL) were superior in the i.v. high-dose betahistine and s.c. betahistine groups relative to sham treatment and the i.v. low-dose betahistine group (Figure 1). Interestingly, in the later stages of vestibular compensation (from days 15 to 30 post-UL), the p.o. betahistine/selegiline group and the s.c. betahistine group were still superior to sham treatment with a 5- to 10-fold higher movement distance, which was even above the baseline level before UL. With regard to these results, it seems likely that the constant release of betahistine through the osmotic pump (39) might be more effective than the pulsatile application of betahistine twice a day. Due to the previously reported anxiolytic effects of selegiline, it is possible that selegiline did not only enhance the effect of betahistine as an MAO-B-inhibitor but also added to more active locomotion by lowering the anxiety of the animals in the open field (40, 41). It is remarkable that betahistine effects on static compensation were rather mild and acute, while effects on mobility were found both in the acute and to a higher extent in the long-term course, despite drug application at day 1 to 3 post-UL only. There are two potential explanations for the predominant effect of betahistine on locomotor activity: (1) a specific effect of betahistine on mechanisms of dynamic vestibular compensation. One might speculate that the application of betahistine in the vulnerable early phases of vestibular compensation leads to an amplification of movement even after the cessation of treatment. Recent studies indicate that an increase in locomotor activity is a consistent feature of dynamic vestibular compensation (27, 42); (2) a non-specific (i.e., non-vestibular) effect of betahistine on locomotor behavior. Alvarez et al. showed a dose-dependent increase in locomotor activity by betahistine in animals without a vestibular lesion, possibly by the modulation of attention and avoidance behavior (43). In contrast to the stable trend toward a higher movement distance in our study until day 30 post-UL, the previously reported effects of betahistine in control animals tended to diminish over time during repetitive application. Irrespective of its specific origin, the augmentation of locomotor activity by betahistine may have a benefit for the long-term recovery from a vestibular lesion, as opposed to medications used for symptom control only such as antiemetics or sedatives, which may rather hamper the long-term outcome.

From a clinical perspective, the dynamic aspects of vestibular compensation and recovery seem to be functionally as important for the mobility and quality of life of patients with acute unilateral vestibulopathy as the static aspects. This is especially true, as patients with acute unilateral vestibulopathy often move less due to vertigo/dizziness and unsteady gait, which further worsens their symptoms. In summary, our findings suggest that presumed higher peaks and reduced fluctuations of betahistine plasma levels result in a better recovery of locomotor and postural symptoms of UL in the acute and chronic phases of vestibular compensation.

### “Target engagement” of betahistine in central vestibular networks

Several mechanisms have been hypothesized to explain the therapeutic effects of betahistine on symptom recovery after UL. Functionally, betahistine is a weak agonist to histamine H1 receptors and an antagonist to histamine H3 receptors, which act as autoinhibitors of histamine release and autoregulators of histamine synthesis (16, 44, 45). Betahistine thus increases the synthesis and release of histamine by blocking histamine H3 autoreceptors and other transmitters by blocking H3 heteroreceptors, which may result in a restoration of bilateral neuronal activity between the vestibular nuclei by a post-synaptic action at various receptors (46, 47). Indeed, H1 expression is increased in the ipsilesional GABAergic commissural neurons located in the medial vestibular nucleus after UL in rats (14). An excitation of vestibular commissural GABAergic neurons on the lesion side would help to rebalance the bilateral vestibular nuclei by the inhibition of the contralesional side (29, 48, 49). In fact, selective blockage of the H1 receptor attenuates the therapeutic effects of betahistine for vestibular compensation in the UL rat model (14).

In the current study, we consistently found an increase in regional cerebral glucose metabolism in the ipsilesional vestibular nucleus on days 1 and 3 post-UL, which was dose-dependent and exactly paralleled the time course of improvement of postural asymmetry. The effects on rCGM in the ipsilesional vestibular nucleus were most prominently seen for i.v. high-dose betahistine and p.o. betahistine/selegiline treatment (Figures 3–5). The activation pattern is compatible with an increase in neuronal activity in the ipsilesional vestibular nucleus, which may counteract the initial decrease following the vestibular lesion (30). Based on the limited spatial resolution of μPET, we cannot differentiate between effects in the medial or lateral vestibular nuclei. An increase in rCGM could therefore reflect either a higher neuronal activity of inhibitory commissural (GABAergic) or excitatory non-commissural neurons, both of which would result in rebalancing of bilateral neuronal activity in the vestibular nuclei. Interestingly, the effects of betahistine on metabolic rates in the ipsilesional vestibular nuclei were found only until day 3 post-UL. By this time, previous [^18^F]-FDG-μPET studies have shown a normalization of rCGM in the vestibular nuclei to baseline level (24, 30). In addition, a previous rat study found that the overexpression of H1 receptors in the ipsilesional medial vestibular nucleus also peaked at 1–4 days post-UL (14). It, therefore, seems likely that the histaminergic effect of betahistine at the level of the vestibular nuclei relies on the microenvironment of asymmetric firing rates and overexpression of H1 receptors in the acute phase of the vestibular syndrome. Interestingly, an increase in the regional glucose metabolism projecting on the vestibular commissural projections was again found at day 30 post-UL in the i.v. high-dose betahistine and p.o. betahistine/selegiline groups. This may correspond to delayed effects in the later stages of vestibular compensation. Similar findings have been made in MRI-based studies in patients following vestibular neuritis (50). The whole-brain [^18^F]-FDG μPET imaging approach indicated further betahistine-related effects on rCGM outside the vestibular nucleus area: (1) A bilateral increase of neuronal activity in the posterior and lateral thalamic nuclei was found consistently from day 1 to 30 post-UL in the groups treated with i.v. high-dose betahistine and p.o. betahistine/selegiline. This effect likely reflects an augmentation of mechanisms of thalamic multisensory integration (51). Both the visual and the somatosensory systems play a key role when it comes to substitution and compensation for the loss of vestibular function (52, 53). Increased thalamic neuronal activity was reported recently for other drugs with a positive effect on vestibular compensation (26, 27). Higher thalamic glucose metabolism evolved in parallel with the increased mobility in the groups with presumed higher betahistine plasma levels. The persistence of thalamic activity argues against a direct histamine receptor-mediated effect of betahistine at the thalamic level. (2) Some moderate increase in glucose metabolism was found in the ipsilesional vestibulocerebellum with a peak 1–3 days post-UL in the i.v. high-dose betahistine group compared to the sham treatment group. The vestibulocerebellum has been implicated in vestibular compensation (30). We did not find changes in glucose metabolism at the hypothalamic level (i.e., the tuberomammillary nuclei), as has been suggested by previous *in vitro* experiments in cats (18, 54).

Taken together, this study provided evidence for a dose-dependent central “target engagement” of betahistine at the level of the ipsilesional vestibular nucleus and vestibulocerebellum during the acute stage of vestibular compensation and a secondary effect on thalamic nuclei involved in multisensory integration. An additional effect at the inner ear level, for example, by the improvement of microcirculation (55, 56) cannot be excluded, but does not seem to be the main mechanism of betahistine action in our UL rat model.

### Implications for future clinical trials and use

The current clinical approval of betahistine for vestibular disorders (mostly Menière's disease and acute unilateral vestibulopathy) is restricted to a daily total dose of 48 mg/day p.o. This recommendation dates back to the market entry of betahistine in the late 1960's. However, this mode of application may lead to variable plasma levels by interindividual differences in absorption and metabolism by MAO enzymes. Based on this notion, a more individual titration and dose escalation of betahistine was propagated in recent years (21). However, randomized controlled trials with 3 × 48 mg per day oral betahistine failed to prove a significant effect compared to placebo in Menière's disease (BEMED trial) (57) and in acute unilateral vestibulopathy (BetaVest trial) (58). Currently, the most promising strategies for further drug development are the combined administration of betahistine and MAO-B inhibitors such as selegiline or rasagiline (59), the parenteral application of betahistine via an intranasal route, and the application of higher betahistine doses and slow-release formulations. Given the extensive dose range of betahistine, with a reported LD_50_ of ~3,000 mg/kg p.o. or 500 mg/kg i.v. in rats, it is highly unlikely that these strategies will result in harmful plasma concentrations. Based on clinical experience, higher doses of betahistine are well-tolerated with headaches being the most prevalent side effect due to the vasodilatory effects of betahistine.

### Strengths and limitations

The strengths of the current study are the direct comparison of different doses and application protocols for betahistine in a standardized preclinical model of acute unilateral vestibulopathy, which closely resembles the clinical syndrome of vestibular neuritis/acute unilateral vestibulopathy, and the use of *in vivo* whole-brain imaging to document the drug action in critical vestibular hubs within the central nervous system. The approach of PET-based visualization of drug-target engagement in the central nervous system is established in the literature (60). However, we agree that the rCGM changes cannot be interpreted as proof of drug action on a functional level. Changes in regional glucose metabolism do not allow further statements on the exact mode of action at receptor levels. A further limitation of the current study is that plasma levels of betahistine were not analyzed and could therefore not have been compared between application protocols. Parameters from behavioral testing in the open field (such as locomotor activity) may be hard to interpret in isolation as a surrogate for dynamic vestibular compensation without other metrics (e.g., rearing measurement and rotarod testing). Future studies need to include combined test measurements of balance and gait control to disentangle the origin of the increased locomotor activity reported as the main effect of this study.

## Conclusion

Betahistine has therapeutic potential for the augmentation of vestibular compensation if the administration is optimized toward higher effective plasma levels. This can be achieved by higher drug doses, inhibition of the MAO-based metabolism, or a parenteral route. In future, the “one-size-fits-all” concept for betahistine administration needs to be revised, and more reasonable strategies for dose-finding have to be considered.

## Data availability statement

The raw data supporting the conclusions of this article will be made available by the authors, without undue reservation.

## Ethics statement

The animal study was reviewed and approved by Regierung von Oberbayern.

## Author contributions

MA: data analysis and interpretation, design of figures, and drafting of the manuscript. ML, EE, LG, CB, AK, and RB: execution of experiments, data analysis, and review of the manuscript. AD: PET data analysis and review of manuscript. RO: execution of experiments. MW: statistical data analysis and interpretation and review of the manuscript. SZ and MS: planning of experiments, data interpretation, and review of the manuscript. AZ: project idea, planning of experiments, data analysis and interpretation, and drafting of the manuscript. All authors contributed to the article and approved the submitted version.
