# Supplementary material for: Dose- and application route-dependent effects of betahistine on behavioral recovery and neuroplasticity after acute unilateral labyrinthectomy in rats
Source: Front Neurol. 2023 Jul 19;14:1175481. doi: 10.3389/fneur.2023.1175481 (PMC10395078; doi:10.3389/fneur.2023.1175481)
Supplement: Supplementary file 1 [file Data_Sheet_1.PDF]

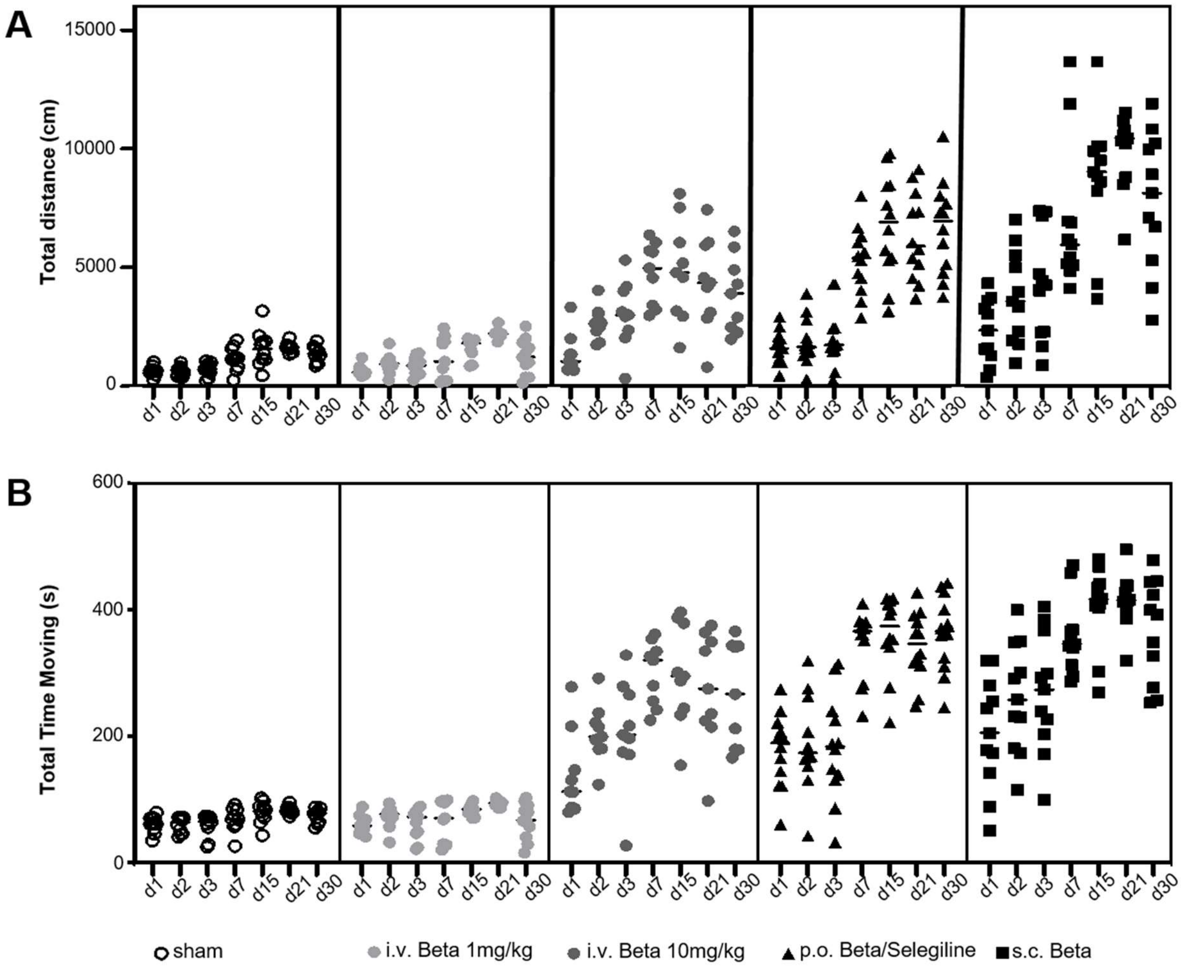

Supplementary Figure 1: Locomotor parameters in the open field (individual values). A) Total amount of the distance moved (in cm) per rat in the open field. **B)** Cumulative duration of movement (in s) per rat. Beta: betahistine, d: day, cm: centimeters, s: seconds.

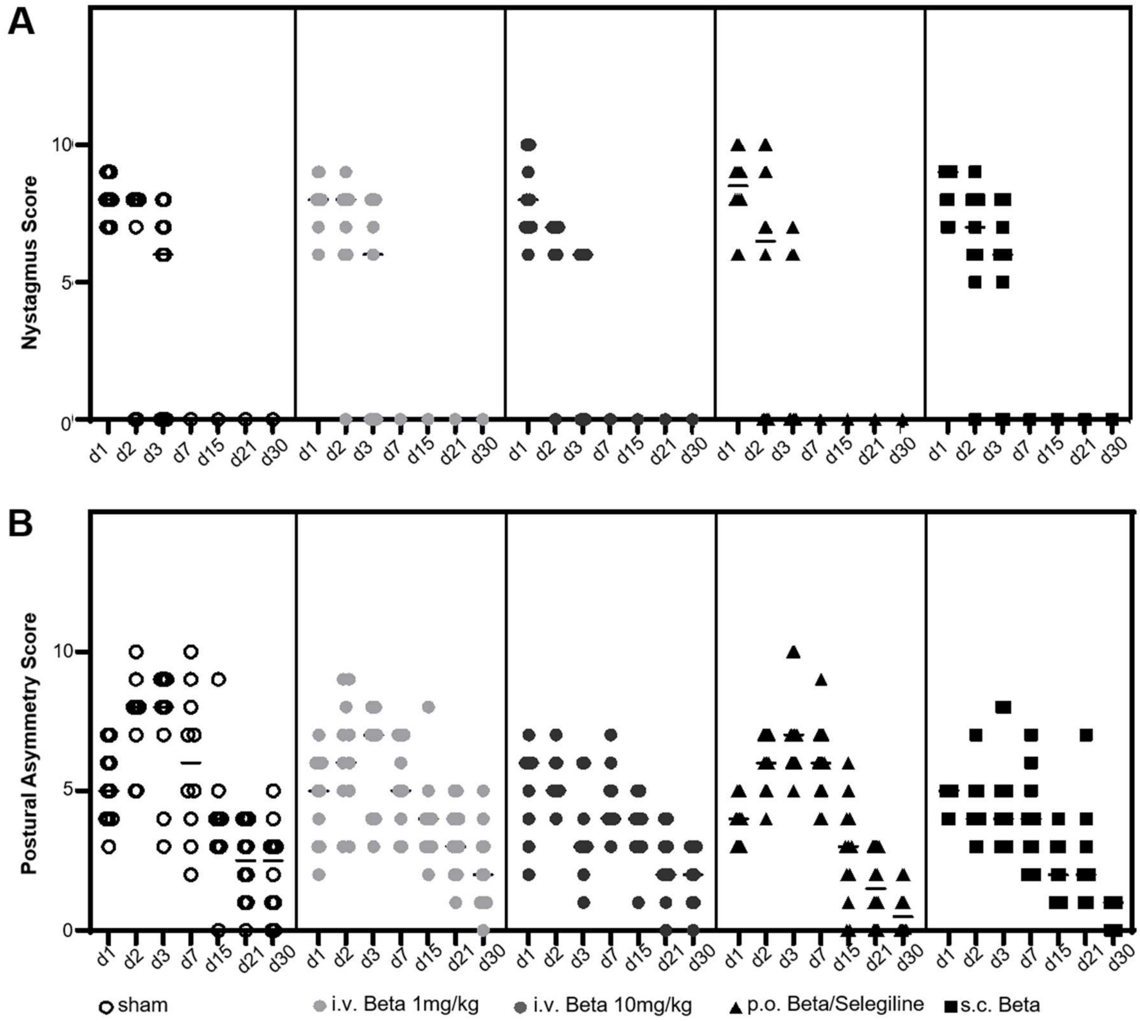

Supplementary Figure 2: Clinical scoring of nystagmus and postural asymmetry post UL (individual values). A) Nystagmus score. **B)** Postural asymmetry score. Beta: betahistine, d: day,
